# Recommendations to advance digital health equity: a systematic review of qualitative studies

**DOI:** 10.1038/s41746-024-01177-7

**Published:** 2024-06-29

**Authors:** Sarah Wilson, Clare Tolley, Ríona Mc Ardle, Lauren Lawson, Emily Beswick, Nehal Hassan, Robert Slight, Sarah Slight

**Affiliations:** 1 School of Pharmacy, Newcastle University, Newcastle Upon Tyne, UK; 2 Translational and Clinical Research Institute, Newcastle University, Newcastle Upon Tyne, UK; 3 Trinity College Dublin, Dublin, Ireland; 4 The Newcastle upon Tyne Hospitals NHS Foundation Trust, Newcastle Upon Tyne, UK

**Keywords:** Public health, Social sciences

## Abstract

The World Health Organisation advocates Digital Health Technologies (DHTs) for advancing population health, yet concerns about inequitable outcomes persist. Differences in access and use of DHTs across different demographic groups can contribute to inequities. Academics and policy makers have acknowledged this issue and called for inclusive digital health strategies. This systematic review synthesizes literature on these strategies and assesses facilitators and barriers to their implementation. We searched four large databases for qualitative studies using terms relevant to digital technology, health inequities, and socio-demographic factors associated with digital exclusion summarised by the CLEARS framework (Culture, Limiting conditions, Education, Age, Residence, Socioeconomic status). Following the PRISMA guidelines, 10,401 articles were screened independently by two reviewers, with ten articles meeting our inclusion criteria. Strategies were grouped into either outreach programmes or co-design approaches. Narrative synthesis of these strategies highlighted three key themes: firstly, using user-friendly designs, which included software and website interfaces that were easy to navigate and compatible with existing devices, culturally appropriate content, and engaging features. Secondly, providing supportive infrastructure to users, which included devices, free connectivity, and non-digital options to help access healthcare. Thirdly, providing educational support from family, friends, or professionals to help individuals develop their digital literacy skills to support the use of DHTs. Recommendations for advancing digital health equity include adopting a collaborative working approach to meet users’ needs, and using effective advertising to raise awareness of the available support. Further research is needed to assess the feasibility and impact of these recommendations in practice.

## Introduction

The World Health Organisation (WHO) advocates Digital Health Technologies (DHTs) to advance population health^1^. Digital health can be defined as the use of information and communication technologies within healthcare to provide healthcare users with services relating to the prevention, detection, diagnosis and management of diseases and other health conditions^2–4^. Examples of DHTs include smartphone applications and wearable monitoring devices that can empower people to better manage their own conditions, such as keeping track of symptoms or remotely monitoring their condition(s) over time^2–4^. DHTs can pick up signs of deterioration in healthcare users’ symptoms longitudinally and provide real-time data to healthcare professionals to help support tailored clinical decision making^4^. DHTs can also enable individuals with mobility issues and those living in rural areas to access healthcare. Digital health has gained global momentum due to its potential to contribute to personalised health care for patients, improved quality of care, and lower healthcare costs^5,6^.

However, there are growing concerns that DHTs may not lead to health benefits in all populations, with underserved groups (i.e., those typically left out of research or experience inadequate access to healthcare) at particular risk^7^. One possible factor contributing to this is digital exclusion, denoting disparities in motivation, access and use of DHTs across different demographic groups^8^. Digital exclusion can potentially create a barrier for various underserved groups, such as those who are on a low income, are not fluent in English, or homeless, thus exacerbating health inequities for these groups^9^. Individuals with visual impairment may also find on-screen reading challenging and many older adults with hearing impairments have expressed low motivation to use phone calls as a remote option to access healthcare due to their disability^10^.

Technology has advanced rapidly over recent years, with some DHTs (e.g., telehealth services, mobile phones, wearable devices, smartphone apps and other software) having greater relevance to the direct inequities underserved groups face compared to other DHTs. For example, DHTs designed to be solely used by healthcare professionals (e.g., electronic patient records) are less likely to directly impact healthcare service users, and so it is prudent to focus on DHTs that underserved groups may be asked to use. Qualitative studies gathering rich in-depth experiences from those whose voices are rarely heard (i.e., underserved groups)^11,12^ will provide valuable insights into the facilitators and barriers regarding access, motivated and/or use of DHTs.

The WHO Bellagio eHealth Evaluation Group (2019) recognised the need to mitigate digital exclusion^13^, with organisations such as NICE (National Institute for Health and Care Excellence) requiring evidence that health inequities have been considered in the design of DHTs^2^. This includes important aspects of design, development or implementation of a DHT that support digital inclusivity, such as strategies to increase an individual’s access to suitable devices or connectivity, and educational support in digital literacy to increase DHT use^14^. To support the development of such strategies, it is vital to understand the needs of underserved groups as well as their experiences and perspectives of these strategies to identify what does and does not support digital inclusivity. However, there is currently no qualitative systematic review of key strategies conducted in this area; a key knowledge gap in the literature. To advance digital health equity, we aimed to systematically synthesise the literature on what key strategies have been used to promote digital inclusivity, and assess the facilitators and barriers to implementing and adopting these in practice based on underserved groups’ experiences and perspectives.

## Results

### Study descriptions

**Fig. 1:**
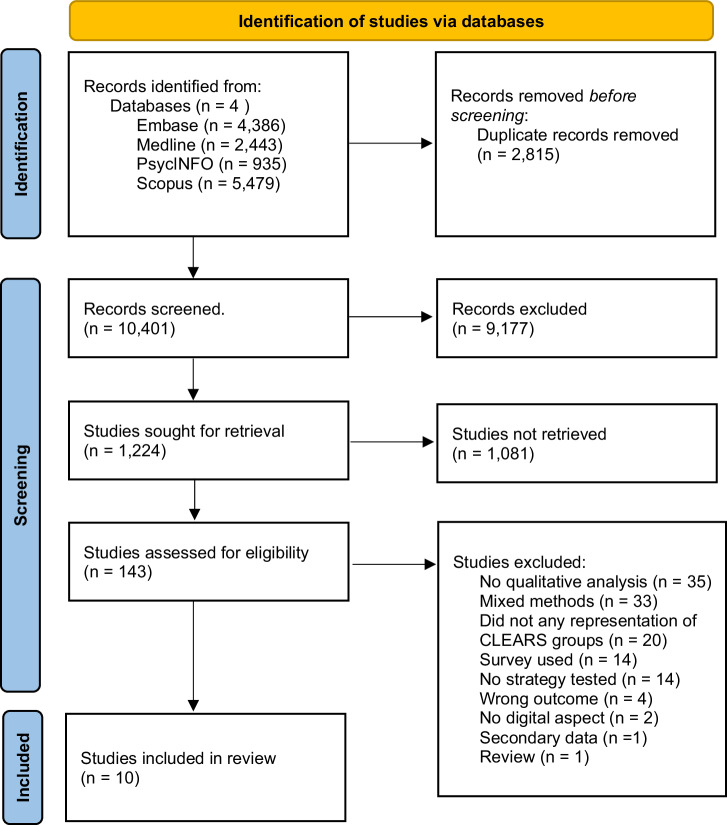
Prisma flowchart. A PRIMSA flow chart detailing our search and selection process applied during the article screening process.

Our search yielded 13,216 results. After removing duplicates (*n* = 2815), titles (*n* = 10,401) abstracts (*n* = 1224) and full-texts (*n* = 143) were screened. Ten papers met our inclusion criteria (Fig. 1). Inter-reviewer reliability was high with 99.33% agreement at title stage, 99.43% at abstract stage, and 97.89% at full-text stage. All included studies were found to have moderate- to high-quality levels (Supplementary Tables 7 and 8). None of the included studies measured or reported any participants’ health literacy.

Included studies incorporated a range of participants at risk of digital exclusion, including those from different cultural backgrounds (ethnic diversity, languages and religion) (*n* = 8)^15–23^, those with limiting conditions (visual and hearing impairments) (*n* = 2)^21,22^, low educational attainment (*n* = 4)^15,19–21^, aged over 65 (*n* = 4)^16,20–22^, homeless (*n* = 2)^19,24^, and those who had low socioeconomic status (*n* = 5)^15–18,21^ (Supplementary Table 9). All 10 studies used interviews^15–24^, with two studies also conducting focus groups with participants^18,21^. (Supplementary Table 10). Inclusive digital health strategies were grouped into either outreach programmes providing educational support and/or access to devices (*n* = 2)^19,22^, or co-designing DHTs with underserved groups (*n* = 8) to gain feedback on the usability and acceptability of DHT to enhance inclusivity in future versions of the DHT (Table 1)^15–18,20,21,23,24^.

**Table 1:** Summary of inclusive digital health strategy explored in the included studies

Inclusive digital health strategy	Study	Population and type of support (outreach) / technology (codesign) explored
Outreach Programme	Howells et al.^19^	Homeless individuals’ experiences of nurses and community support workers to promote their access and use of DHTs in response to COVID-19.
	Mizrachi et al.^22^	The role of family members in supporting online health services usage amongst older adults (+65 years).
Co-design Approach	Kramer et al.^18^	Use of an embodied conversational agent (an interactive digital healthcare professional avatar) designed to encourage trust in healthcare systems and professionals amongst ethnically diverse groups.)
	Yeong et al.^21^	Specifically designed health website to improve accessibility for those with visual impairments.
	Maidment et al.^20^	Specially designed smartphone app to aid those with hearing impairment use hearing aids.
	Asgary et al.^24^ Kim et al.^17^	Experiences of homeless individuals^24^ and linguistically ethnically diverse individuals with low educational attainment and low income^17^ using mHealth (the use of mobile or smartphone devices for health purposes).
	Alkureishi et al.^15^ Wikaire et al.^23^	Experiences and opinions of those with low educational attainment, on a low socioeconomic income^15^ and ethnically diverse individuals^15^^,^^23^ when using general digital health services to explore how to support use, access and motivation.
	Choxi et al.^16^	Experiences and opinions of ethnically diverse older adults with a low socioeconomic status on video consultations to explore how to better support their use of this technology.

A table detailing the inclusive digital health strategies explored in each of the ten included studies.

**Fig. 2:**
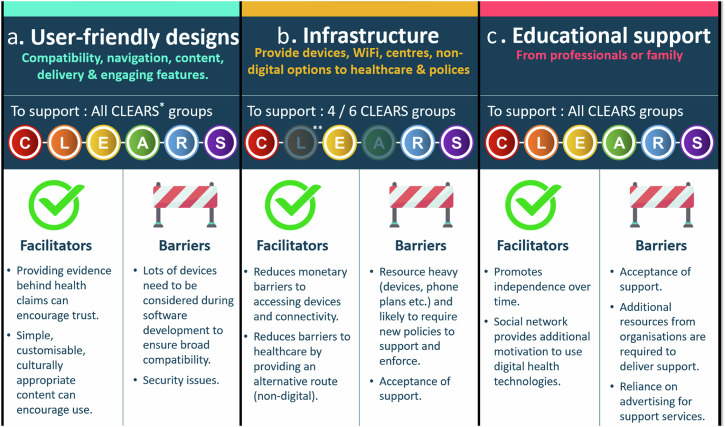
Facilitators and barriers of the three key strategies to support digital health equity. Summary of the key facilitators and barriers to strategies to support digital health equity (using a user-friendly design, providing infrastructure and providing educational support) alongside the specific CLEARS groups the strategy will support.

Our narrative thematic synthesis generated three overarching themes; user-friendly designs (e.g., software and website design elements that promoted inclusivity), infrastructure (e.g., access to DHTs) and educational support (e.g., training to develop digital literacy skills required to use DHTs) (Supplementary Table 10). Facilitators and barriers to the adoption of these themes are embedded in the discussion below and summarised in Fig. 2.

#### User-friendly designs

User-friendly designs were a key theme supporting access and use of DHTs across seven studies^15,16,20–22,24^. Health-related software and websites needed to be compatible across different digital platforms, operating systems and devices including smartphones and desktops, and assistive technologies (e.g., screen reading software) to accommodate the needs of ethnically and linguistically diverse groups^17,18,22^, individuals with limiting conditions (visual and hearing impairments)^20,21^, older adults (+65 years)^20,22^, those with low educational attainment and low socioeconomic status^17,18^. For example, Yeong et al. noted how older adults with visual impairments and of low socioeconomic status needed websites to be compatible with different magnification levels and assistive technologies (e.g., iOS Voiceover [Apple Inc]; a screen reading software) to aid visibility^21^. The authors also noted how certain features aided navigation and minimised scrolling to help the user find information, such as tables of contents, drop-down menus, and ‘jump to top’ buttons^21^. Older adults with limiting conditions (visual or hearing) also suggested that navigation features, such as search bars and hyperlinks, needed to be of high contrast (compared to the rest of the screen) to improve visibility^20,21^. Yeong et al. emphasised how search features should be designed in a similar way to commonly visited search engines, like Google, to improve usability and reduce confusion^21^.

Older adults, homeless, ethnically diverse individuals and those with visual impairments all described how digital messages on software, health related websites or text messages should be simple, concise, and presented in a logical manner without time restrictions^18,20,21,24^. For example, older adults with visual impairments described how they did not have enough time to read the information when presented on a timed loop (i.e., rotating between different screens with information), and suggested that the user be able to manually control the timing of this loop^21^. Older adults interviewed in another study described how it would be useful if they could change the font size to improve the visibility of the text, and provide alternative languages for those who are not fluent in English^22^. Personalising information, such as allowing users to choose content that they are interested in, was felt to be one way of increasing the motivation to use health related websites and software amongst those with visual impairments^21^ and ethnically diverse individuals^18^. Additionally, providing evidence that supported the key messages in healthcare information, such as the importance of reducing alcohol intake to reduce the risk of developing chronic health conditions, enhanced trust amongst ethnically diverse individuals^18^. Kramer et al. also emphasised how any communication should be culturally appropriate and avoid reinforcing stereotypes, especially for ethnically and linguistically diverse users^18^. For example, the language used to categorise different ethnicities on DHTs should avoid generic terms such as *‘men of colour’* as some ethnically diverse men found this offensive; they felt it defined them based on their skin colour and not their ethnic background. Instead, specific terminology should be used that accurately represented their ethnicities (e.g., African American for individuals with an African and American descent)^18^. Any imagery should also be inclusive to all cultural groups^18^.

It was felt that the overall user friendliness and engagement of health related software could be improved with the addition of engaging features^15,18,20,22,24^. This included interactive quiz elements^20^, notifications encouraging behavioural changes^18^, reminders about upcoming appointments (particularly for homeless individuals as this they may not have access to other reminders, like letters)^24^, ability to order a repeat prescription and schedule specific appointments (e.g., physiotherapy)^22^. Older adults of Jewish faith also suggested simplifying security features, as many found flicking between a text message with the password reset information and the screen (where the information should be entered) challenging^22^.

#### Infrastructure

Five studies described the need for supportive infrastructure, such as access to devices and connectivity (i.e., Wi-Fi) to support homeless individuals, ethnically and linguistically diverse groups, and individuals of low socioeconomic status^15,17,19,23,24^. For example, Howell et al. explained how community nurses in the UK provided homeless individuals with temporary access to smartphones during the pandemic so as to enable them to access vital digital healthcare support^19^. In the United States (US), homeless individuals were provided with phones (the Obama phone), credit and data plans financed through a government programme^24^. However, Asgary et al. found that some of these homeless individuals using the Obama phone plan often exceeded their limits when put on hold to schedule medical appointments^24^. They subsequently turned to friends and family for financial support to purchase credit^24^. Other homeless individuals were hesitant to accept this government support, with the authors reflecting on how this may have been due to the homeless experiencing a lack of government financial aid in the past^24^.

Homeless individuals^19^, ethnically and linguistically diverse groups^15,17,23^, and those of low educational attainment and low socioeconomic status^15,17,19^ reported relying heavily on free Wi-Fi to be able to access healthcare. This included accessing free Wi-Fi in public spaces and transport systems, fast-food restaurants, clinics and families’ houses. However, they often experienced barriers to this connectivity with time limits set by the specific organisations (e.g., opening hours)^15^ or restrictions placed on using shared devices (e.g., computer keyboards due to the risk of coronavirus spreading)^19^. Many participants suggested creating dedicated centres for digital health services with suitable devices and free Wi-Fi that would also include some private areas^15^. Access to these private spaces was felt to be important for some ethnic and linguistically diverse groups with low educational attainment and socioeconomic status, as they were concerned about being overheard when discussing/looking at confidential health information^17^. Many groups suggested that they would like the choice between both digital and non-digital access to healthcare, as this would help mitigate the risk of possibly excluding those with poor digital literacy skills, those who would prefer in-person consultations, or those who lack the resources to access digital healthcare^15,18,19,23^.

To complement infrastructural changes, ethnically diverse adults based in the US advocated for more resources to be provided by local government^15^. This included the introduction of new policies, such as reduced payment plans and regulations on the price of DHTs for lower income earners to make them affordable^15^. Older adults of Jewish faith and ethnically diverse adults with a low educational attainment and socioeconomic status also suggested that financial incentives could help promote greater access to DHTs and encourage motivation to use DHTs^15,22^. Alkureishi et al. highlighted how different organisations, such as hardware and Wi-Fi companies, might need to collaborate to ensure that these different components (e.g., devices, connectivity, financial aid) are jointly available to support successful implementation^15^.

#### Educational support

Provision of educational support was important for ethnically diverse individuals and older adults to enable their use of DHTs in five studies^15,16,19,20,22^. Ethnically diverse individuals with lower educational attainment and low socioeconomic status, and older adults of Jewish faith commonly reported asking family members to remain close during video healthcare consultations in case of technical issues^15^, or for their guidance with accessing online health information^22^. Mizrachi et al. found this support promoted independence over time as older adults’ digital skills developed through learning and they were further motivated to use DHTs on hearing positive experiences from their family and friends^22^.

Some individuals relied on educational support from professional services to use DHTs^19^. It was felt that in-person educational support from community workers or health care professionals with supplementary materials (e.g., videos and written information) would be beneficial prior to attending virtual appointments to support ethnically diverse adults (both above and below 65 years) from a low socioeconomic status and low educational attainment^15,16^ Alternatively, Alkureishi et al. noted some participants expressed preference for accessing training classes at healthcare sites (e.g., hospitals) and community centres, where support was provided by ‘technology champions and coaches’^15^. However, older adults of Jewish faith highlighted how advertisements to promote awareness of support services would be unlikely to reach individuals in their community and those who were socially isolated and arguably most in need of support^22^. Some studies also highlighted how certain groups (e.g., ethnically diverse adults with low socioeconomic status and low educational attainment, and older adults of Jewish faith) might also be reluctant to accept this educational support due to concerns around burdening others, feeling helpless, and/or reaffirming how they are unable to do something independently^15,22^.

## Discussion

This systematic review synthesises strategies that promote digital inclusivity and assess the barriers and facilitators to adopting these in practice. Our findings highlighted three key themes relating to user-friendly designs, supportive infrastructure, and provision of educational support. Barriers to adopting these strategies included a lack of acceptance amongst some underserved groups to receive such support, whilst facilitators included promoting trust amongst ethnically diverse groups by providing lay term friendly evidence that supports health claims.

Our findings highlighted how health-related software and websites must be interoperable across different devices to accommodate the needs of underserved groups. This form of user-friendly design is advocated by national healthcare providers and government bodies; for example, the UK and US have legislation in place which mandates that websites and software in the public sector be *‘perceivable, operable, understandable and robust’* to ensure that those with visual and hearing impairments, low reading ability (reading age of 9) and/or those who are not fluent in English can access and understand the information provided^25,26^. However, a recent study reported that public health authority websites in only three countries (UK, Italy, China) out of a total of 24 actually adhered to these accessibility standards when checked^27^. Additionally, the wider literature supports our findings on how the use of appropriate language and imagery can improve end-user satisfaction^18,28^. National bodies, such as the US National Institute of Health (NIH), have developed the ‘National Culturally and Linguistically Appropriate Services (CLAS) Standards’ to assist developers and researchers in developing culturally and linguistically appropriate services^29^. The wider literature also suggests co-designing DHTs with underserved groups at the earliest stages to help ensure that they meet the needs of all end-users^30^. This involves co-designing security features that are easy-to-use and align with the UK government ‘*secure by design principles’*, to help overcome any potential future barriers to usage^31,32^.

Our results also highlighted the need for supportive infrastructure to facilitate access and use of DHTs. Government schemes in high-income countries are already available; for example, the ‘Obama phone’ in the USA and the Emergency Broadband Benefits and social tariffs (reduced payment phone plans) in the UK, to support those on a low income to access smartphones and phone plans^24,33^. However, implementing supportive infrastructure might not be viable for low to middle income countries as they may have less suitable centres to provide devices and free public Wi-Fi spots, which high income countries already have access to^34^. Some charity organisations, such as the Good Things Foundation, have started to repurpose donated corporate IT devices and deliver them to those who are digitally excluded^35^. However, better promotion of the support available and a collaborative working environment is needed, especially by healthcare professionals, social services, and charities. Free phone numbers would also help to facilitate access to healthcare services. Some underserved groups would like the option of accessing healthcare via non digital means, thus questioning the temptation to always use technology to potentially address healthcare challenges^36^. Researchers need to consider whether a new DHT will provide an equitable solution to the healthcare problem and whether other means of accessing healthcare should also be provided within healthcare systems^37^.

This systematic review also underlined the importance of providing educational support, from family or professional services, to encourage motivation and capability to use DHTs. There is a need for effective advertising of this support to groups at particular risk of both digital and social exclusion, such as older adults and homeless individuals, in order to increase their awareness^38^. A systematic review conducted by Ige et al.^39^ suggested using a combination of two or more strategies to reach socially isolated individuals, including referrals from relevant agencies (e.g., GPs, pharmacists etc), as this might be a more effective approach than relying solely on public facing methods^39^.

**Fig. 3:**
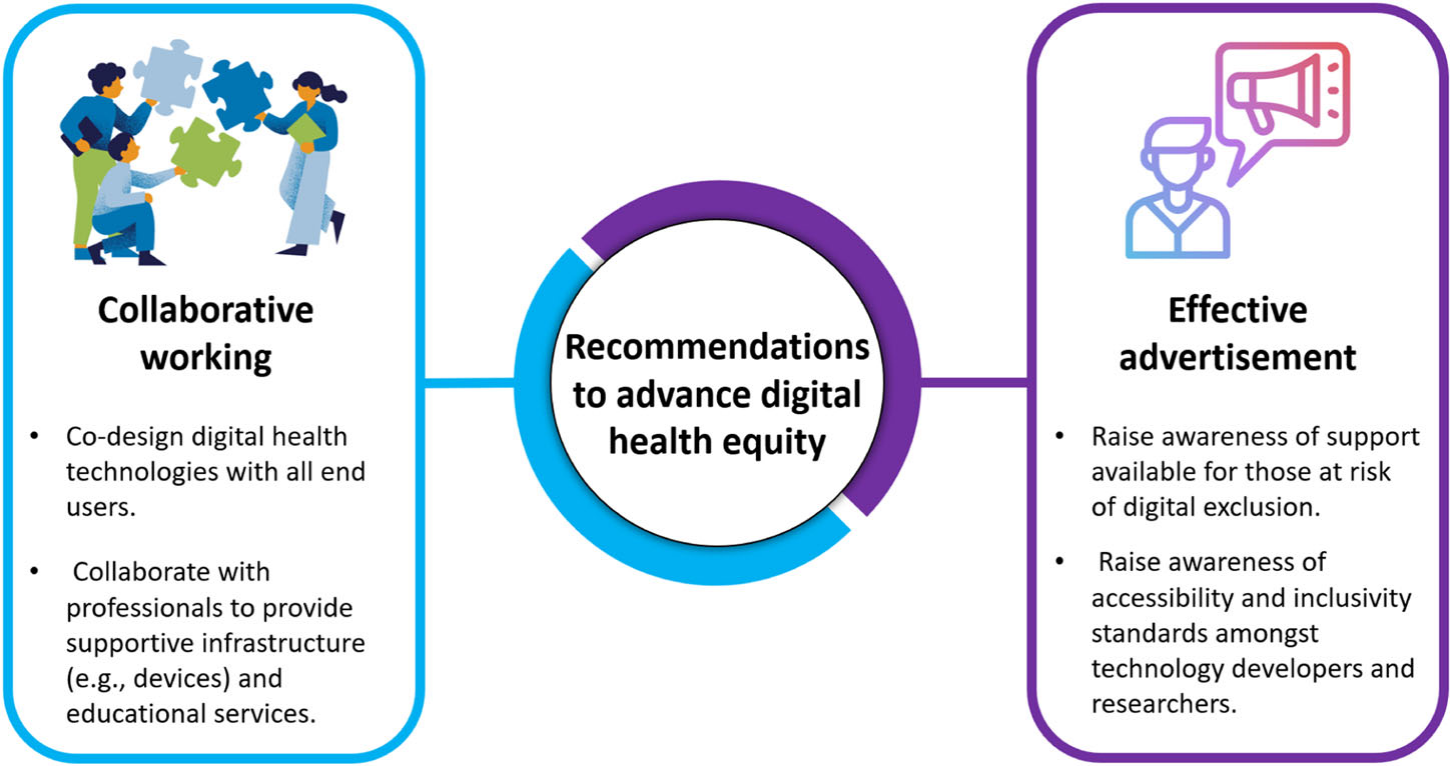
Key recommendations for the design, development and implementation of key strategies which make a digital health intervention inclusive. Summary of the two key recommendations to advance digital health equity, centring around adopting a collaborative working environment and using effective advertisement.

Previous recommendations to promote digital health equity have centred around guidance for behavioural and social science researchers with limited insight to the facilitators and barriers to implementing strategies into society and appear limited to research settings^40^. Previous reviews have applied the socioeconomic model to inform recommendations to promote digital health equity, such as providing devices (individual level support), educational support (relationship/interpersonal level support), access to connectivity infrastructure (community level support) and implementing policies (societal level support)^41,42^. However, there has been little consideration given to those individuals who belong to two or more underserved groups at risk of digital exclusion. Our systematic review considered this intersectionality and provides practical recommendations that focus on two main areas: collaborative working and effective advertising (Fig. 3). Collaborative working between the DHT developer, healthcare professionals, policy-makers, voluntary sectors, patients and public members of underserved groups is vital to help improve the co-design of DHTs and provision of support and should be embedded from the very beginning of the design and development process^30^. Effective advertising strategies are also vital to raise public awareness and ensure that those who are, or know of an individual, at risk of digital exclusion are made aware of in-person support that is available and how to access it. DHT developers and researchers should also be aware of the accessibility and inclusivity standards (e.g., government legislation and CLAS) and on how to use them to support digital health equity.

This review used a comprehensive and systematic approach to identify relevant literature. Included studies were published within the last decade to remain relevant to the current digital healthcare landscape. We opted to focus on qualitative research to gather rich detailed information on the facilitators and barriers to each strategy. Despite no geographical restrictions being placed on this search, we found that all included studies were conducted in high-income countries, which may limit the applicability of these findings to low- and middle-income countries; this also highlights the importance of further work in this area. Representation of the different religious groups and languages was limited, highlighting a gap in the literature and a need for greater diverse inclusion in research. None of the included studies reported on participants’ health literacy, which has previously been suggested to overlap with low digital literacy^43^; this information would have aided our understanding of whether the participants included in the qualitative studies were truly representative of the groups that they were intended to represent. Future research should incorporate a standardised health literacy measure, such as the Newest Vital Sign (NVS)^44^ or the Health Literacy Questionnaire (HLQ)^45^, into their methodology to provide greater detail on the participants in their study.

The appropriateness of recommendations from this systematic review could be further explored using an established framework, such as the APEASE criteria (Affordability, Practicability, Effectiveness, Acceptability, Size effects/safety, and Equity)^46^. This would involve seeking the perspectives of CLEARS demographic groups’ and relevant stakeholders’ (e.g., policy makers and community workers) on the practicalities of implementing these different strategies and recommendations to further advance this important area of digital health equity. The facilitators and barriers to implementing government-issued public health website accessibility standards should also be explored to further understand how to encourage use of these standards.

This systematic review identified three key themes relating to digital inclusivity, associated facilitators and barriers, and recommendations for advancing digital health equity. This information will guide individuals when designing, developing and implementing digital health interventions to ensure it is done in a digitally inclusive manner. This review also highlighted the need for further work to explore the feasibility and acceptance of implementing different strategies and recommendations to support digital health equity amongst those at risk of digital exclusion.

## Methods

### Identification of key groups at risk of digital exclusion

**Fig. 4:**
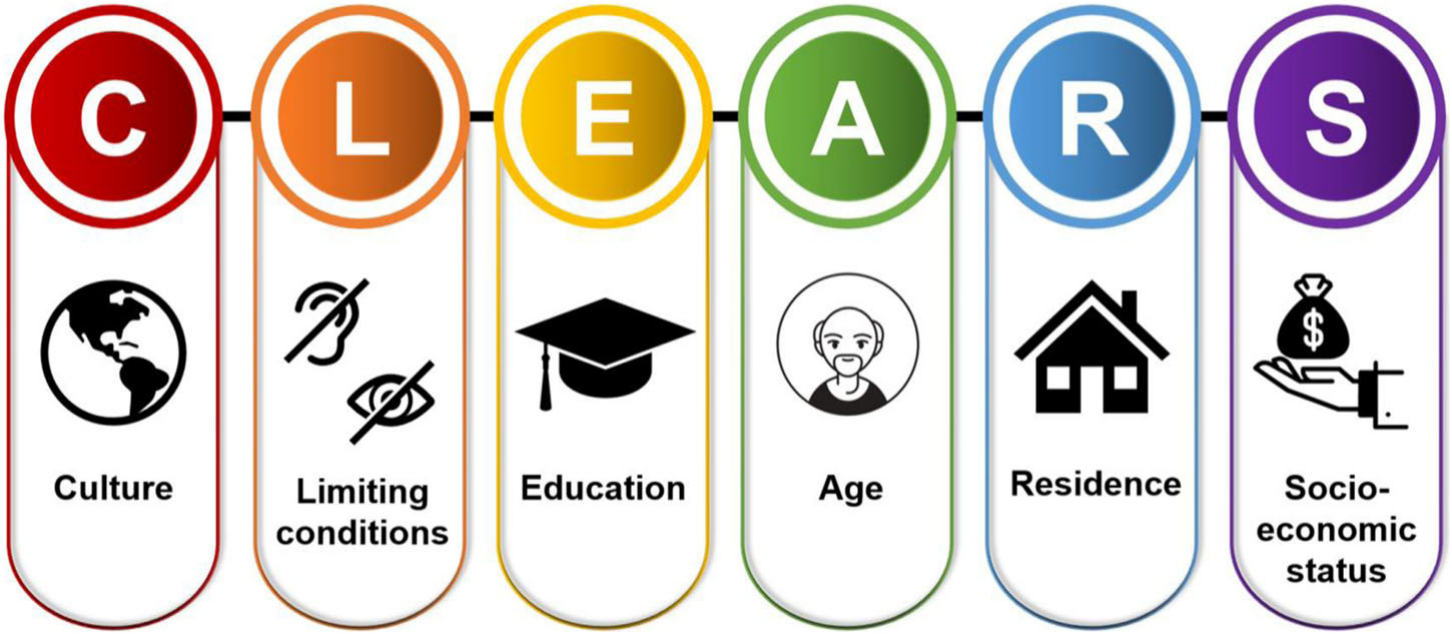
Groups of sociodemographic factors that are at risk of digital exclusion within healthcare. A framework which encompasses sociodemographic factors associated with digital exclusion and recognises the role of intersectionality.

We conducted a scoping review of the literature to identify the sociodemographic factors that could put an individual at risk of digital exclusion. Based on the findings published in peer-reviewed articles^24,47–60^, systematic reviews^61–64^, government reports^8,65^, and regulatory organisation documents^66^, we identified a number of sociodemographic factors that we complied into six groups, relating to Culture (ethnicity, language, and religion)^8,47–53,62^, Limiting conditions (visual and hearing impairments)^6,54,66^, Education (at or below United Kingdom (UK) government mandated level or equivalent)^52,55,56,66^, Age (over 65 years)^51,54–56,62,66^, Residence (rural or deprived areas [based on consensus data within a country], or homeless)^8,24,51,60^, and Socioeconomic status (low income [earns less than 60% of the median household annual income within a country] and unemployed individuals)^8,52,55,56,62,65,66^ abbreviated to CLEARS (Fig. 4). These factors often intersect (i.e. intersectionality), placing an individual at even greater risk of digital exclusion^8,64–66^.

### Search strategy

This systematic review was registered with PROSPERO (CRD42022378199) and followed PRISMA guidelines^67^. The search string utilised terms from two relevant scoping reviews^8,68^, with additional relevant terms included when searching four large online databases (Medline, Embase, PsycINFO and Scopus) (Supplementary Tables 1–4). The search focused on words associated with digital technology, health inequities, and CLEARS (Table 2).

**Table 2:** Summary of eligibility criteria

	Inclusion	Exclusion
Population	Studies the six groups categorised within CLEARS.	Studies which focused solely on children (under 18 years old) and/or did not include any of the six groups within CLEARS.
Intervention	Studies focusing on inclusive digital health strategies to promote CLEARS groups access to general healthcare though DHTs’ designed for healthcare service users.	Studies which did not focus on inclusive digital health strategies, studies that focusing on using DHT to deliver care for one specific aspect of health such as sexual health, nutrition, maternal health, children’s health or substance misuse, and/or DHT’s designed to be used by healthcare professionals such as electronic patient records.
Outcome	Studies discussing facilitators or barriers associated with inclusive digital health strategies for CLEARS groups.	Studies that did not discuss any facilitators or barriers.
Study design	Qualitative methods only (including interviews, focus groups and/or observations).	Quantitative or mixed methods.
Language	Studies published in the English language.	Studies not published in the English language.
Date of publishing	Studies published between 2012 and 2022.	Study published before 2012.
Publication type	Primary research peer-reviewed journal articles.	Grey literature, reviews, letters to the editor, conference abstract or proceedings or posters, study protocols and journal articles that have not been peer reviewed.

A table detailing our inclusion and exclusion criteria applied to article screening process.

### Eligibility criteria

The eligibility criteria followed the Population, Intervention, Comparison, Outcomes and Study design (PICOS) framework, recommended by the Cochrane Handbook for Systematic Reviews^69^, and provided an organising framework to list the main concepts in the search. The Population criteria included any group represented by our CLEARS framework (see above). The Intervention criteria focused on inclusive digital health strategies, which we defined as an action designed to alleviate the digital exclusion of individuals by promoting access, motivation, and/or use of information and communication technologies^2–5^. Articles needed to have discussed the facilitators or barriers associated with the inclusive digital health strategy (outcome criteria) to be included. This allowed the researchers to reflect on what currently worked or did not work to inform key recommendations. Only qualitative studies that provided rich in-depth experiences from CLEARS groups were included to aid our understanding of how a complex phenomenon, i.e., intersectionality, can affect digital exclusion^11,12^. Quantitative studies were excluded as they are designed to test a hypothesis or enumerate events or phenomena^11,12^, which was not aligned with the aim of this review. Only peer-reviewed articles published between 2012 and 2022 in the English language were included; this ensured only the latest advancements in digital technologies were considered.

### Study selection

Results from each database were exported into EndNote (version 20.5, Clarivate, International) and duplicates removed. Remaining articles were uploaded to Rayyan (Qatar Foundation, State of Qatar)^70^, where titles, abstracts, and full-texts were screened independently by two reviewers (SW, LL, EB) to minimise bias. The lead author (SW) screened all articles, acting as a constant throughout the process. Disagreements were resolved by a third reviewer (RMA). The reasons for excluding full text articles were recorded (Fig. 1).

### Data extraction and synthesis

The lead author (SW) developed a data-extraction sheet with the research team to extract and record specific study details, including participant demographics and a description of the inclusive digital health strategy under investigation (Supplementary Tables 5 and 6). Any measure used to record participants’ health literacy in the included studies, such as the Newest Vital Sign (NVS)^44^ or the Health Literacy Questionnaire (HLQ)^45^, was also extracted. A quality assessment was carried out on the included studies using the Critical Appraisal Skills Programme (CASP) Qualitative Review Checklist^71^. Quality was measured by reporting the frequency of ‘yes’ (denoting the study met the criteria on the checklist) (Supplementary Tables 7 and 8).

The lead author (SW) performed a narrative thematic synthesis of the included studies. Firstly, the authors began by developing a preliminary synthesis of findings from included studies to identify the key strategies and list the facilitators and barriers to implementation. We then considered the factors that might explain any commonalities and differences in the successful implementation of these digital inclusive strategies across included studies. This involved exploring the directly reported verbatim quotations obtained from particular CLEARS groups and seeking to draw descriptive and explanatory conclusions around key themes^72,73^. All data management and analysis was carried out within N-Vivo (version 1.6.1, QSR International). Discussions with co-authors (SPS, RM, CT) were conducted at several stages throughout the analysis to discuss, refine and define themes to ensure a coherent narrative that reflected the data. Detailed descriptions and contextual material from the included studies was kept throughout the analysis to ensure that the trustworthiness was upheld^74,75^. Ethics approval was not required for this systematic review.

### Reporting summary

Further information on research design is available in the Nature Research Reporting Summary linked to this article.

### Supplementary information

Supplementary Information

Reporting Summary

## Data Availability

All relevant data used for the study has been included in the manuscript and supplementary information.
