# Supplementary material for: Recommendations to advance digital health equity: a systematic review of qualitative studies
Source: NPJ Digit Med. 2024 Jun 29;7:173. doi: 10.1038/s41746-024-01177-7 (PMC11217442; doi:10.1038/s41746-024-01177-7)
Supplement: Supplementary file 1 — Supplementary Information [file 41746_2024_1177_MOESM1_ESM.pdf]

Supplementary table 1: Search strategy used in Embase

**Category**	**Search string**
CLEARS groups	exp ancestry group/ or exp ethnicity/ or exp "ethnic or racial aspects"/ or exp ethnic group/ or BAME.mp./ or exp cultural factor/ or exp religion/ or exp English as a second language/ or language.mp./ or exp visual impairment/ or exp hearing impairment/ or exp educational status/ or exp adult/ or exp residence characteristics/ or exp rural population/ or exp rural area/ or exp homeless person/ or exp developing country/ or exp rural health care/ or medically underserved area.mp./ or exp socioeconomics/ or exp income/ or exp health insurance/ or exp sociodemographics/ or exp minority group/ or exp vulnerable population/ AND
Inequities	exp health care disparity/ or exp racial disparities/ or exp health care access/ or health inequities.mp./ or exp health disparity/ or exp digital divide/ or digital inequities.mp./ or digital exclusion.mp./ or digital gap.mp/ or exp urban rural difference/ or underserved.mp. or exp medically underserved/ or underrepresented.mp./ or exp computer literacy/ or exp internet access/ AND
Digital technology	exp Internet/ or exp "internet use"/ or exp digital technology/ or exp telehealth/ or ehealth.mp./ or exp algorithm/ or exp mobile application/ or exp smartphone/ or "technology use".mp./ or exp remote sensing/or exp wireless communication/ or exp wearable computer/ or wearable technology.mp./ or smart home.mp./ or exp telemedicine/ or digital health technologies .mp./ or patient portal.mp./ or mhealth.mp./ or exp "internet of things"/ or apps.mp.

Key: exp = exploded mesh term, .mp. = key word search. A limit was placed on the search to only include papers indexed as the publication type "article", published between 2012- 2022 and are in the English language. This search was conduced 25/11/2022.

Supplementary table 2: Search strategy used in PsycInfo

**Category**	**Search string**
CLEARS groups	exp "Racial and Ethnic Groups"/ OR exp Ethnic Identity/ OR BAME.mp./ OR exp Sociocultural Factors/ OR exp Religion/ OR language/ OR exp language proficiency/ OR exp Hearing Disorders/ OR exp Vision Disorders/ OR exp Educational Attainment Level/ OR adult.mp./ OR exp Rural Environments/<br>OR exp Homeless/ OR exp Developing Countries/ OR exp Rural Health/ OR exp Socioeconomic Factors/ OR exp Deprivation/ OR exp Poverty Areas/ or exp Poverty/ OR exp Middle Income Level/ or exp Lower Income Level/ OR exp Health Insurance/ OR exp Demographic Characteristics/ OR exp Minority Groups/AND

Inequities	exp "Race and Ethnic Discrimination"/ OR exp Health Disparities/ OR exp Cultural Sensitivity/ OR exp "Racial and Ethnic Differences"/ OR exp Racial Disparities/ OR exp Health Care Access/ OR health inequities.mp./ OR exp Digital Divide/ OR digital inequities.mp./ OR digital exclusion.mp./ OR digital gap.mp./ OR urban rural divide.mp./ OR exp Disadvantaged/ OR underserved.mp./ OR underrepresented.mp./ OR exp Digital Literacy/ OR internet access.mp./ AND
Digital technology	exp Digital Technology/ OR exp Wireless Technologies/ OR exp Wearable Devices/ OR exp Smartphones/ OR remote sensing technology.mp./ OR exp/ OR Algorithms/ OR smart home.mp./ OR telehealth.mp./ OR exp Telemedicine/ OR digital health technology.mp./ OR exp Electronic Health Services/ OR ehealth.mp./ OR exp Mobile Applications/ OR exp Computer Usage/ OR technology usage.mp./ OR internet of things.mp./ OR exp Electronic Health Records/ or patient portal.mp./ OR mhealth.mp. OR exp Mobile Health/ OR apps.mp./

Key: exp = exploded mesh term, .mp. = key word search. A limit was placed on the search to only include papers indexed as the publication type “article”, published between 2012- 2022 and are in the English language. This search was conduced 25/11/2022.

Supplementary table 3: Search strategy used in Medline

**Category**	**Search string**
CLEARS groups	exp racial groups/ OR exp ethnicity/ OR exp "Ethnic and Racial Minorities"/ OR BAME.mp./ OR exp cross-cultural comparison/ or exp cultural characteristics/ OR exp Religion/ OR exp Language/ OR exp limited english proficiency/ OR exp persons with hearing impairments/ or exp visually impaired persons/ OR exp Educational Status/ exp Adult/ OR exp Residence Characteristics/ OR exp Rural Population/ OR exp Homeless Persons/ OR developing contries.mp./ OR exp Rural Health Services/ OR exp Medically Underserved Area/ OR exp Socioeconomic Factors/ OR exp Income/ OR exp health benefit plans, employee/ or exp insurance, medigap/ or exp medicare/ OR exp sociodemographic factors/ OR exp Minority Groups/ OR exp "health disparity, minority and vulnerable populations"/ AND
Inequities	exp Healthcare Disparities/ OR exp Health Services Accessibility/ OR exp Health Inequities/ OR exp Digital Divide/ OR digital exclusion.mp./ OR digital inequities.mp./ OR digital gap.mp./ OR urban-rural divide.mp. / OR underserved.mp./ OR underrepresented.mp./ OR exp Computer Literacy/ OR exp Digital Technology/ OR exp Remote Sensing Technology/ AND

Digital technology	Wireless Technology/ OR wearable technology.mp. or exp Wearable Electronic Devices/ OR exp Telemedicine/ or smart home.mp./ OR digital health technology.mp. or Mobile Applications/ OR exp Smartphone/ OR ehealth.mp./ OR telehealth.mp./ OR exp algorithms/ OR racial disparities.mp./ OR exp Internet Access/ OR exp "Internet Use"/ or exp Internet/ or exp "Internet of Things"/ OR exp Internet-Based Intervention/ OR apps.mp./ OR mhealth.mp./ OR exp Patient Portals/

Key: exp = exploded mesh term, .mp. = key word search. A limit was placed on the search to only include papers indexed as the publication type "article", published between 2012- 2022 and are in the English language. This search was conduced 25/11/2022.

Supplementary table 4: Search strategy used in Scopus

**Category**	**Search string**
CLEARS groups	ethnicity OR "racial groups" OR "ethnic identity" OR bame OR religion OR language OR "sociocultural factors" OR "hearing impairments" OR "visual impairments" OR "educational attainment" OR adult OR "residence characteristics" OR rural OR homeless OR "developing contr*" OR "rural health" OR "medically underserved area" OR socioeconomic OR deprivation OR poverty OR income OR "health insurance" OR "sociodemographic factors" OR "minority groups" OR "vulnerable populations" AND
Inequities	"healthcare disparit*" OR "race and ethnic discrimination" OR "racial disparit*" OR "healthcare access" OR "health inequit*" OR "digital divide" OR "digital inequit*" OR "digital exclu*" OR "digital gap" OR "urban rural divide" OR disadvantage OR underserved OR "digital literacy" OR "internet access" OR underrepresented AND
Digital technology	internet OR "internet use" OR "digital technolog*" OR "wireless technolog*" OR "wearable technolog*" OR smartphone OR "mobile app*" OR "remote sensing technolog*" OR algorithm OR "smart home" OR telehealth OR telemedicine OR "digital health technolog*" OR ehealth OR "technology use" OR "internet of things" OR "digital health technolog*" OR "patient portal" OR mhealth OR apps AND

Key: exp = exploded mesh term, .mp. = key word search. A limit was placed on the search to only include papers indexed as the publication type "article", published between 2012- 2022 and are in the English language. This search was conduced 25/11/2022.

Supplementary table 5: Table a: Data extraction form focusing on study characteristics.

Authors	Year	Aim	Method	CLEARS	Other demographic factors	Sample size	Location (i.e. Country)
Alkureishi, M.A., Choo, Z.-Y., Rahman, A., Ho, K., Benning-Shorb, J., Lenti, G., SÃ¡nchez, I.V., Zhu, M., Shah, S.D., Lee, W.W.	2021	Interview patients to understand their perspectives on (1) the definition, causes, and impact of the digital divide; (2) whose responsibility it is to address the digital divide; and (3) potential solutions to mitigate the digital divide.	Interviews	Hispanic (2), black/ African American (23), white (8), other (2). Private medical insurance (10), Medicare (25). High school or less (14). All reported English as their primary language	Adult primary care patients (35/54), and 19 were parents of paediatric patients who had a phone, video or both types of visits at a Chicago medical centre. Adult sample: Hispanic (2), black/ African American (23), white (8), other (2). Female (27), male (8). Private medical insurance (10), Medicare (25). High school or less (14). All reported English as their primary language	54 (35 participants adult & of focus to the aims, the other 19 were parents of patients)	Centres for Medicare and Medicaid Services (CMS) - Chicago (USA)
Asgary, R. And Sckell, B. And Alcabes, A. And Naderi, R. And Adongo, P. And Ogedegbe, G.	2015	To evaluate homeless individuals perceptions, attitudes and experiences regarding mobile health	Interview	Homeless	Average Age (SD; Range): 51.66 (±11.34; 25 to 79), Female: 29 (58%), Age above 50: 33 (66%), Average years of homelessness (SD): 2.03 yrs (SD±3.10), max 14 yrs, History of Chronic Dis. 30 (60%), Active mental illness 10 (20%)	50	New York City (USA)
Kim, H., Zhang, Y.	2015	Exploring the context in which smartphones were used for health information.	Interview	Hispanic, low education, low income	20 low-SES adults self-identified as Hispanics in Texas: Females (12), males (8), 18 had an annual income of less than $25,000, 15 had only high school or less than high school education, age ranged from 20 to 60 (Mean¼34.6; SD¼ 13.8), 15 participants spoke both English and Spanish, with the majority (13 out of 15) speaking Spanish as the first language, 11 reported having some kind of health-related concerns.	20	Texas (USA)
Kramer, Justin and Yinusa-Nyahkoon, Leanne and Olafsson, Stefan and Penti, Brian and Woodhams, Elisabeth and Bickmore, Timothy and Jack, Brian W.	2021	Black men's experiences with, and suggestions for, health care systems, while also exploring the acceptability of ECA technology (embodied conversational agent (ECA) that simulates face-to-face conversation with a health counsellor) to address some of these concerns	Focus groups and interviews	Ethic diverse men, some spoke Spanish, some low education, some unemployed	Focus groups: perceptions & experiences of healthcare, factors affecting health behaviour change, acceptability of ECA model.<br>Boston (3 groups, 8 ppt):<br>• mean age: 23.4<br>• Ethnicity: black African American (5), black other (3)<br>• Education: high school (4), uni (4)<br>• Employment: full time (2), part time (4), unemployed (1), student (1)<br>Providence (1 group and 7 ppt excluded as minors, include 3 groups and 19 ppt):<br>• mean age: 21.6<br>• Ethnicity: black African American (14), black other (5)<br>• Spanish speaking at home (3)<br>• Education: no high school (4) less than high school (6), high school (4) uni (5)<br>• Employment: full time (2), part time (3), unemployed (1), student (3)<br>New Haven (1 group and 12 ppt excluded as minors, include 1 groups and 12 ppt):<br>• mean age: 24<br>• Ethnicity: black African American (11), black other (1)<br>• Spanish speaking at home (3)<br>• Education: no high school (1) less than high school (3), high school (4) uni (4)<br>• Employment: full time (3), part time (9), unemployed (0), student	67 in focus group and 5 in interviews (12 key informant interviews excluded, Remove 19 as minors (under 18)).	Boston (MA), New Haven (CT), Providence (RI), and Philadelphia (PA) with programs serving young Black and African American men. (USA)

					(0)<br>Philadelphia (2 groups, 9 ppt):<br>• mean age: 19.8<br>• Ethnicity: black African American (8), black other (1)<br>• Education: less than high school (4), high school (2) uni (3)<br>• Employment: full time (0), part time (1), unemployed (3), student (5)<br>Interviewees (N = 5) were recruited from the sample of 30 men who used ECA for 2 weeks.<br>• ages of 18 and 27 years, mean age of 19.4.		
Maidment, D.W., Heyes, R., Gomez, R., Coulson, N.S., Wharrad, H., Ferguson, M.A.	2020	Compare barriers and facilitators between m2Hear and C2Hear	Interviews	(1) adults aged ≥18 years, (2) adults who had never worn hearing aids, (3) adults who were familiar with smartphone technologies, and (4) adults who had a good understanding of the English language to understand the content. Exclusion criteria included those who were unable to use m2Hear unassisted due to cognitive decline or dementia, determined via a self- or familial report. (Limiting condition - hearing & Age - most over 65)	1st time hearing aid users recruited through a medical centre in Nottingham<br>• Female (6) male (10)<br>• Age range: 39-85 mean: 68.81<br>• 2 self-reported as a beginner in digital tech competency and 14 as fully competent	16	Nottingham (UK)
Mizrachi, Y. And Shahrabani, S. And Nachmani, M. And Hornik, A.	2020	Provide a qualitative picture of these invisible obstacles and to profile their main features, with special attention to the role of family members in supporting OHS use among this population group.	Interviews	The proportions of Jews and non-Jews were similar. Moreover, non-users and users were also represented in proportion to the survey findings, as were age and gender and other relevant demographic variables. In terms of age, 12 respondents were between the ages of 50 and 54, six respondents were between the ages of 55 and 60, seven respondents	People picked from the sample the authors used in a quantitative survey in a different study<br>• proportions of Jews (82% reported in questionnaire) and other religion (18%)<br>• 21 users – 9 men and 12 women; 10 non-users – 4 men and 6 women<br>• ages between 50 and 54 (n=12), 55 and 60 (n=6), 61 and 69 (n=7), 70 and above (n=6)	31	Israel

				were between 61 and 69, and six respondents were 70 years old and above.			
Wikaire, E. And Harwood, M. And Wikaire-Mackey, K. And Crengle, S. And Brown, R. And Anderson, A. And Jansen, R. M. And Keenan, R.	2022	Investigated Māori experiences of Telehealth consultations during the March 2020 COVID-19 lockdown. Participants were asked about what worked, what did not work, and for suggestions to improve future Telehealth provision to Māori whānau	Interviews - A Māori interviewer employed te reo (Māori language), tikanga (Māori protocols) and whanaungatanga (Māori customary engagement rituals) to facilitate the interview process.	Native new Zealand people	Little info given	14 ppt: 5 Māori health professionals (will exclude), 6 Māori telehealth consultation patients, and 6 Māori in-clinic consultation patients.	New Zealand
Yeong, J. L. And Thomas, P. And Buller, J. And Moosajee, M.	2021	Identify key web-based accessibility features for internet users with vision impairment and to explore whether the contents provided in website were relevent and comprehensible.	Observation & focus group	1 above 65 (Ages 25-70), , Visually impaired (n=6), controls(n=3) based in London, 1 unemployed, 2 retired;	4 females, 5 male, 1= 25, 1= 37, 3= 40-50, 2= 50-60, 1= 62, all use internet, all at least somewhat confident in using tech . Some clinicians also texted but it doesn't give much info on them.	9	Moorfields Eye Hospital NHS Foundation Trust, London (UK)
Howells, K. And Amp, M. And Burrows, M. And Brown, J. And Brennan, R. And Dickinson, J. And Jackson, S. And Yeung, W. L. And Ashcroft, D. And Campbell, S. And Blakeman, T. And Sanders, C.	2022	Explore the experience and impact of organisational and technology changes in response to COVID-19 on access to health care for people experiencing homelessness.	Interviews	Homeless , Ethnicity: white British (18), mixed race (2), eastern European (1), Education: no qualifications (11), GCSE (8), degree (1), missing (1)	• 3 females; 18 males<br>• Age ranges: 25-34 (n=5), 35-44 (n=6), 45-54 (n=8), 55-64 (n=2)<br>• Ethnicity: white British (18), mixed race (2), eastern European (1)<br>• Education: no qualifications (11), GCSE (8), degree (1), missing (1)<br>• All have at least one chronic condition<br>• All reported anxiety & depression and/ or alcohol & drug related issues	21 (22 excluded as they are social workers/ clinicians)	North West England (UK)
Choix et al	2022	Identify the barriers of video visits for patients who rely on telephone visits for ambulatory care.	Interviews	Ethnicity: Majority were Black, Indigenous, and People of Colour (BIPOC) (n=6), Average age: was 68, and a majority (n=5) were 70 or older, Socioeconomic status: Majority had	The average age of the randomly sampled patient participants was 68, and a majority (n = 5, 56%) were 70 or older. A majority were Black, Indigenous, and People of Colour (BIPOC) (n = 6, 67%), and a majority had Medicaid or Medicare (n = 7, 78%). Staff participants included a Care Coordinator (n = 1, 25%), a Medical Assistant Supervisor (n = 1, 25%), a Patient Access Specialist (n = 1, 25%), and a Behavioural Health Resource Specialist (n = 1, 25%).	9 (4 excluded as they were clinical support staff)	Portland, Oregon (USA)

	Medicaid or Medicare (n=7).			

Supplementary table 6: Data extraction form focusing on key information used to inform the analysis.

Authors	Factors causing inequities / barrier the strategy is trying to overcome	Inclusive strategy used	Benefits and Challenges	Key findings / notes
Alkureishi, M.A., Choo, Z.-Y., Rahman, A., Ho, K., Benning-Shorb, J., Lenti, G., SÃ¡nchez, I.V., Zhu, M., Shah, S.D., Lee, W.W.	Telemedicine utilization increased during the COVID-19 pandemic, divergent usage patterns for video and audio-only telephone visits emerged. Older, low-income, minority, and non-English speaking Medicaid patients are at highest risk of experiencing technology access and digital literacy barriers. This raises concern for disparities in health care access and widening of the “digital divide,” While studies demonstrate correlation between racial and socioeconomic demographics and technological access and ability, individual patients’ perspectives of the divide and its impacts remain unclear.	Co-design: Understanding patient views on the digital divide and how they think it could be mitigated.	Providing free resources/wifi - financial burden on healthcare system / gov but relieve financial burden on underserved populations . Provision through libraries / clinics - availability of services and opening times/ lack of knowledge of available resources but allows individuals to access wifi/ tech which they don’t have at home. Low tech modalities - phone/post - interoperability problems but will reach individuals who have no access to wifi/ tech at home/ older generation who don’t want to use tech. Education / tech champions in community - financial burden on community/ gov to pay individuals for the serve	1)understand tech needs: survey to assess resources people need, 2)access to tech: "Free access to Wi-Fi and providing something to access that, like a computer or a tablet. 3) Providing the resource for these people to be able to access. Even libraries are not enough or some small things like that are difficult. [Patient 53]", patient friendly portals that can be integrated with families accounts, use for libraries to provide tech/ wifi. 4) Education - provide educational/ skill building classes, tech support e.g. Tech champions for the community. 5) low tech approaches running parallel i.e. Landline phones, postal mail, home visits to access those who don’t have tech/ wifi at home or can't travel to libraries etc. To get access to resources.
Asgary, R. And Sckell, B. And Alcabes, A. And Naderi, R. And Adongo, P. And Ogedegbe, G.	Little information exists regarding strategies that use mhealth (i.e., mobile technology for providing health information or services) to mitigate barriers to healthcare access among the homeless. Exploring perceptions, attitudes and experiences of the homeless regarding potential mhealth methods may help design programs to mitigate some of these barriers and address health disparities among homeless individuals.	Co-design: previous experiences text messaging healthcare info including appointment reminders, health education, or management of diseases.	Small phones are hard for older generation to type on/ theft can occur / changing no's - longest a person is quoted to keep the same no. Is 3 years, question the possibility of leaving someone out of the loop if they change their no. And don’t tell healthcare/ state provided phone 'Obama phone' was used by most and well liked although the plan didn't provide unlimited minutes leading to some running out - some had distrust in the government to provide a phone plan with more minutes on of free	Phones to provide reminders for appointments were well liked and Obama phones were used by most - unlimited data/ minute plans should be provided to encourage more use of phones for health info. Most liked simple texts with only relevant information rather than lengthy motivational messages. Some prefer phone calls but most preferred a text as they can "can save or store” [F43] it.
Kim, h., zhang, y.	A recent study showed that Hispanics (a group more at risk of health inequities)have the highest smartphone ownership among ethnic groups, and are more likely to look up health information using their phones than blacks and whites (Fox and Duggan, 2013). However, little is known about the users, particularly those of low-socioeconomic status, health information-seeking behaviour using smartphones.	Co-design:	Improve access e.g. Call dr, text, apps, location, bus times, set up dr appointment, quicker to access internet vs computer (log on etc), can access in free time (convenient). Cheaper vs computer. More private than shared computer (e.g. Library).<br>Tech ownership, particularly computers/ UpToDate models, wifi access, limited data plans, small screen hard to read and navigate, some documents not compatible with phone, limited skills (how to use apps, internet and search engines), miss understanding of language (medical jargon, comprehending English if not 1st language, evaluating	Phones are accessible amongst underserved populations but wifi/ data plan could be provided to improve access. Bigger screen phones could be provided or taught how to increase letter size in classes (covering using apps/ internet/ understanding or evaluating health info)

			health i.e. Dr vs google)	
Kramer, Justin and Yinusa-Nyahkoon, Leanne and Olafsson, Stefan and Penti, Brian and Woodhams, Elisabeth and Bickmore, Timothy and Jack, Brian W.	Black individuals are more likely to live in medically underserved areas, which are characterized by an insufficient number of primary care providers, elevated poverty, and poor population health (Molina et al., 2017). Technology may offer an opportunity to address health disparities. The "Gabe System" (hereafter called "Gabe") is an embodied conversational agent (ECA) that simulates face-to-face conversation with a health counsellor - predicted that it could help improve trust of docs if it is culturally sensitive and representative (culturally informed health care, and strategies to promote sustained participant engagement in health care)	Co-design: interviews were with people who had used GABE (interactive avatar)	Focus groups = challenges to underserved communities getting health info - culturally appropriate representation in images and clinicians. A lot of experienced bias / judgment over treatment e.g. Where told they were dumb for fighting and they just wanted their hand fixed / the clinician didn't know the context. Some suggested training clinicians to be culturally sensitive and aware of the issues in their area. With GABE (the cartoon) people were likely to engage with certain content or not was their perception as to how that material may have specific relevance to them (e.g. Student was drawn to sections on alcohol/ drugs) - preference was shared for this to be compatible with phone and get notifications/ set up appointments / get info	ECR are an accepted approach if culturally sensitive and provide relevant info. If compatible with a phone, it was suggested to make it more accessible and could have more features to make it more useful.
Maidment, d.w., heyes, r., gomez, r., coulson, n.s., wharrad, h., ferguson, m.a.	First-time users, for instance, experience difficulties using their hearing aids because they struggle to remember all of the information given to them by their audiologist at the time of fitting [7,8]. Similarly, hearing aid handling skills in existing hearing aid users are highly variable, ranging from poor to excellent [9]. As a result, hearing aids are often used sub optimally or not at all, with estimates of non use varying from 3% to 24% [10]. Unmanaged hearing loss results in persistent psychosocial difficulties that can lead to social withdrawal and isolation for both individuals and their frequent communication partners [11,12]. Although audiological counselling post hearing aid fitting aims to address suboptimal use and non use of hearing aids, information in clinical settings is typically delivered verbally. Consequently, most of the information provided to patients is forgotten or retained incorrectly	Co-design: used an app to help use hearing aid	If someone didn't have a phone they cant use it but people found it was easy to navigate and use. The use of short info chunks and ease navigation met people could find the info they wanted quick and easy. Having the info in an accessible place (on phone) meant people would go back to some info if they had forgot it but would still go to the dr if they thought their hearing had changed or the hearing aid had a fault.	Ease to navigate apps with short chunks of information to allow personalisation was liked. Phone app was perceived as more accessible than computer / using YouTube for info. Falls down in terms of still need a phone & good digital literacy skills to be able to use the tech.
Mizrachi, Y. And Shahrabani, S. And Nachmani, M. And Hornik, A.	Low adoption of OHS amongst +65s. In Israel, people age 65 and above make extensive use of health services (e.g., an average of 11.2 annual visits to general and family physicians compared to an average of only 3.2 annual visits among the general population age 20 and above [3]). Yet according to the 2018 Statistical Abstract of Israel, only 51% of Israelis over the age of 65 use computers, compared to 72% of all adults over the age of 20. Among the	Outreach: role of family members/ younger generation to support tech use	Forgetting password/ security issues e.g. Multi authentication still an issue, most don't have privacy concerns so happy to get family to help and most have family as 1st point of contact if they have a tech issue/ need assistance. Some didn't want family help "i don't need that kind of help" attitude so pride might get in the way / family relations may not be positive to get help. Incentive programs to encourage use were suggested. Font of text needs to increase, reduce or make multi authentication easier, reduce the no. Of times someone needs to change their password & provide language settings to change to the one they prefer. Increase advertising of available services and what is out	Reduced use in old people related to: unawareness of benefit for them personally/ service and not liking looking at screens due to decreased eye sight. Incentive programs to encourage use were suggested. Font of text needs to increase, reduce or make multi authentication easier, reduce the no. Of times someone needs to change their password & provide language settings to change to the one they prefer. Increase advertising of available services and what is out there/ how easy it is to use it. Increase functionality i.e. What services are available to increase usefulness.

	population of older adults who do use computers, the primary uses are information searches (91%), e-mail (72%) and social networks (72%)[4].		there/ how easy it is to use it. Increase functionality i.e. What services are available to increase usefulness.		
Wikaire, E. And Harwood, M. And Wikaire-Mackey, K. And Crengle, S. And Brown, R. And Anderson, A. And Jansen, R. M. And Keenan, R.	Māori experience barriers to accessing timely, quality healthcare. The March 2020 COVID-19 lockdown in New Zealand required provision of Telehealth consultation options in primary care. This scoping project investigated Māori experiences of Telehealth consultations during the March 2020 COVID-19 lockdown.	Co-design: experiences of Telehealth consultations	Time and economic saving (less time waiting to be seen, less time taken off work, no transport costs, easier to look after kids).<br>Made ppt feel in charge of their healthcare (could see times available and pick which suits them, could pick their doctor)<br>Increased flexibility of service and access (improved access to a dr with same cultural background as them & access in remote areas)	Resources (internet access, data, software, tech, info on access to resources was limited)<br>Unmet needs (physical assessments are hard, not all symptoms are acknowledged leading to self-medicating at home, hard to assess some basic symptoms e.g. Temperature if ppt doesn't have thermometer at home)<br>Something new to learn<br>Healthcare systems weren't aiding with financial cost of resources<br>Language (medical terms hard to follow)	Suggestions for improvements: More user-friendly with language options & audio<br>Providing an option of in clinic, over the phone or online<br>Ensuring clinicians are culturally competent & can communicate in a lay term way over telehealth<br>Providing resources needed to use telehealth including education<br>Sense of connection with health care provider – clinicians build an 'online repour' with patient
Yeong, J. L. And Thomas, P. And Buller, J. And Moosajee, M.	Health websites for genetic eye disorders are typically poorly accessible to users with visual impairment	Co-design: usability testing session on a prototype of a accessible website with patients to identify website features that would increase accessibility for users with visual impairment.	Features of the prototype that improve accessibility: Consistent layout (colour theme, size, left aligned); Structured information hierarchy with a clear description of links & use of bullet points; Adaptability to different assistive software & high magnification; Simple home page with easy navigation; Readable content (low reading level / not too detailed)	Features that reduce accessibility<br>• Poor contrast: navigation and search bar were fait and hard to see.<br>• Dynamic content: slides with info that rotate automatically didn't give enough time to read.<br>• Large white spaces: easy to get lost if people use magnifying setting.<br>• Miss understanding how to search (expected google set up but it split the results into 'page title match' and 'page text match'	To make a website accessible to users with visual impairment, attention should be focused on making simple, well-designed pages with consistent layout and information structure, good contrast, and simple navigation, all of which will directly improve the overall user experience. All ppt had basic digital skills - unsure if accessible/ usable amongst those with low digital literacy skills. Sample didn't include anyone using braille keyboard - might affect navigation/ usability.
Howells, K. And Amp, M. And Burrows, M. And Brown, J. And Brennan, R. And Dickinson, J. And Jackson, S. And Yeung, W. L. And Ashcroft, D. And Campbell, S. And Blakeman, T. And Sanders, C.	COVID-19 pandemic has caused unprecedented disruption and change to the organisation of primary care, including for people experiencing homelessness who may not have access to a phone. Little is known about whether the recent changes required to deliver services to people experiencing	Outreach: explore barriers with natural change to remote telephone consultations & community	Improved awareness of help e.g. Individuals right to be registered with a GP. Access increased by community workers helping to make appointments/medical queries on behalf of people experiencing homelessness, supplying pre-paid phones and digital devices to hostels	If worker misses a call if the dr rings back late then this can delay access to patients treatment. The nurses interviewed often went above and beyond their expected	The findings have emphasised the importance of addressing practical and technology barriers as well as supporting communication and choice for mode of consultation. The authors argue that consultations should not be remote 'by default' and instead take into consideration both the clinical and social factors underpinning health. Consultations should not be

	homelessness will help to address or compound inequality in accessing care. Remote access was perceived as time efficient and helped those with anxiety but it did lead to long waiting times to get appointments/ reduced chance of same day / late & missed callbacks delayed access to treatment, reduced access if you don't have funds or access to a phone or computer (covid reduced access to shared computers), tech issues & poor signal reduced quality of communication, makes it harder for the dr to see stuff so ppt felt this reduced good quality diagnosing, reduced personal interaction & empathy.	support workers and clinicians to provide access to primary health care through the pandemic - support works & outreach workers being key facilitators of care.	(such as ipads), to enable easier and timely access, engaging with people to raise awareness of organisational changes, facilitating GP registration, and discussing specific patients as part of a multidisciplinary team meeting within the primary care network. Some facilitating appointments by allowing their patients to use their smartphones.	role and working hours to secure appointments for their patients - adds more workload & pressure to community nurses/ workers involved.	remote 'by default' and instead take into consideration both the clinical and social factors underpinning health.
Choix et al	Due to covid, video visits increased to allow physical examination and aid diagnostic decision making but require video capable technology, reliable internet access and greater digital literacy than phone visits contributing towards inequities.	Co-design: experiences of video consultations to explore how to better support their use of this technology	Video consultation benefits included avoiding unreliable medical transportation and reduced time to complete an appointment. Participants who do not own internet-capable technology are interested in getting assistance to obtain and use it (showing motivation to be more engaged). Highlights the need for supportive infrastructure and educational support.	Some participants reported needing information from providers about why video consultations are better than telephone consultations. Though many have access to technology and use it for tasks such as email, they want in-person help specific to their personal technology to build confidence. Some participants did not have devices compatible for video consultations.	Majority of participants were interested in using video consultations but lacked the confidence to use it or have access to compatible equipment. In-person help was suggested to be the best approach to help individuals.

Supplementary table 7: Section A of CASPA checklist

Authors	Year	1) Was there a clear statement of the aims of the research?	Comment	2) Is a qualitative methodology appropriate?	Comment	3) Was the research design appropriate to address the aims of the research?	Comment	4) Was the recruitment strategy appropriate to the aims of the research?	Comment	5) Was the data collected in a way that addressed the research issue?	Comment	6) Has the relationship between researcher and participants been adequately considered?	Comment
Alkureishi, M.A., Choo, Z.-Y., Rahman, A., Ho, K., Benning-Shorb, J., Lenti, G., SÃ¡nchez, I.V., Zhu, M., Shah, S.D., Lee, W.W.	2021	Y	Stated in abstract	Y	Aimed to understand patient perspectives	Y	Justification for semi structured interviews rather than focus groups is in the discussion	Y	Good detail was given on the setting and sampling strategy	Y	Setting for the data collection was justified, good detail on the development of the interview guide, author noted interviews were digitally recorded, mention of stopping recruitment at data saturation	Y	Good detail is given on researcher characteristics, multiple members were involved in the interview guide development, conducting interviews and analysis reducing bias in the results
Asgary, R. And Sckell, B. And Alcabes, A. And Naderi, R. And Adongo, P. And Ogedegbe, G.	2015	Y	Stated in abstract	Y	Aimed to understand individuals perspectives	Y	Provided justification for conducting interviews and focus groups	Y	Good detail was given on the setting and sampling strategy	Y	It is clear how data were collected & justified the methods chosen, there is an indication of how interviews are conducted, noted that the interviews were recorded and documented verbatim.	N	No detail is given on researcher characteristics or who was involved in the interview guide development or conducting interviews
Kim, H., Zhang, Y.	2015	Y	Stated in abstract	Y	Asked participants to describe their experiences of using smartphones for health information as well as reasons for such usage.	N	Didn't justify way interviews were used over other methods e.g. Focus groups	Y	Good detail was given on the sampling strategy & eligibility screening	Y	It is clear how data were collected & justified the methods chosen, there is an indication of how interviews are conducted, noted that the interviews were audio recorded, mentioned where the interview guide was adopted through the study.	N	No detail is given on researcher characteristics or who was involved in the interview guide development or conducting interviews
Kramer, Justin and Yinusa-Nyahkoon, Leanne and Olafsson, Stefan and Penti, Brian and Woodhams, Elisabeth	2021	Y	Stated in intro	Y	Exploring men's experiences with, and suggestions for, healthcare systems, while also exploring the	N	Didn't justify why they used focus groups and interviews	Y	Good detail was given on the sampling strategy & sample represented people they were focusing on	Y	The setting for the data collection was justified, how the data was collected is clear, the researcher has semi justified the methods chosen 2Because of the exploratory nature of the study and a lack of prior research on	N	No detail is given on researcher characteristics

and Bickmore, Timothy and Jack, Brian W.					acceptability of ECA technology to address some of these concerns						this subject, the semi-structured interview method was adopted", the researcher has made the methods explicit (e.g. Indication of how interviews were conducted, and their topic guide), information was provided about where further questions were asked to get more detail,		
Maidment, D.W., Heyes, R., Gomez, R., Coulson, N.S., Wharrad, H., Ferguson, M.A.	2020	Y	Stated in abstract	Y	Aimed to gain an in-depth insight into the views of the barriers and facilitators	N	Didn't justify way interviews were used over other methods e.g. Focus groups	Y	Good detail was given on the sampling strategy & exclusion criterial to ensure sample represented people they were focusing on	Y	The setting for the data collection was justified, it is clear how data were collected, the researcher has made the methods explicit (e.g. For interview method, is there an indication of how interviews are conducted and topic guide in appendix), the form of data is clear	N	No detail is given on researcher characteristics
Mizrachi, Y. And Shahrabani, S. And Nachmani, M. And Hornik, A.	2020	Y	Stated in abstract	Y	To gain insights regarding the central barriers associated with OHS adoption among older adults in Israel	N	Didn't justify way interviews were used over other methods e.g. Focus groups	Y	Mentions sample was picked from a sample previously used in a connected study & proportionally representative of the original sample	Y	If the setting for the data collection was justified, it is clear how data were collected, the researcher has made the methods explicit (e.g. For interview method, is there an indication of how interviews are conducted, and info on their topic guide)	N	No detail is given on researcher characteristics
Wikaire, E. And Harwood, M. And Wikaire-Mackey, K. And Crengle, S. And Brown, R. And Anderson, A. And Jansen,	2022	Y	Stated in intro	Y	Aim to understand peoples personal experiences	Y	Provided justification for the method	Y	Good detail was given on the sampling strategy & sample represented people they were focusing on	Y	If the setting for the data collection was justified, it is clear how data were collected, the researcher has justified the methods chosen, the researcher has made the methods explicit (e.g. There is an	Y	The research is led and carried out by Māori health researchers (same ethnicity as participants)

R. M. And Keenan, R.											indication of how interviews are conducted), the form of data is clear		
Yeong, J. L. And Thomas, P. And Buller, J. And Moosajee, M.	2021	Y	Stated in abstract	Y	Identify website features that would increase accessibility for users with visual impairment.	Y	Further discussions were prompted by asking participants to expand their answers and seek opinions or counter-opinions from the rest of the group --> good justification for why interviews weren't used.	N	Details on setting but didn't mention how they recruited	Y	The setting for the data collection was justified, it is clear how data were collected, the researcher has justified the methods chosen, the researcher has made the methods explicit (e.g. For interview method, is there an indication of how interviews are conducted), the form of data is clear	N	No detail is given on researcher characteristics
Howells, K. And Amp, M. And Burrows, M. And Brown, J. And Brennan, R. And Dickinson, J. And Jackson, S. And Yeung, W. L. And Ashcroft, D. And Campbell, S. And Blakeman, T. And Sanders, C.	2022	Y	Stated in abstract	Y	Explore the experience and personal impact	Y	Provided justification for the method	Y	Good detail was given on the sampling strategy & sample represented people they were focusing on	Y	The setting for the data collection was justified, it is clear how data were collected, the researcher has justified the methods chosen, the researcher has made the methods explicit (e.g. For interview method, is there an indication of how interviews are conducted, the form of data is clear (e.g. Audi recordings)	Y	The interviews with people experiencing homelessness were led by a researcher employed by Groundswell (a homeless charity) following a peer-research approach. Researchers with lived experience of homelessness receive research skills training (via Groundswell) to enable them to engage with the target research population more easily.
Choix et al	2022	Y	Stated in intro	Y	Explored people's perceived on barriers to video visits	N	Didn't justify why they didn't use focus groups instead	Y	Good detail was given on the sampling strategy & sample represented people they were focusing on	Y	It is clear how data were collected & , there is an indication of how interviews are conducted, mentioned where the interview guide w	N	No detail is given on researcher characteristics

Supplementary table 8: Section B of CASPA checklist

Authors	7) Have ethical issues been taken into consideration?	Comment	8) Was the data analysis sufficiently rigorous?	Comment	9) Is there a clear statement of findings?	Comment
Alkureishi, M.A., Choo, Z.-Y., Rahman, A., Ho, K., Benning-Shorb, J., Lenti, G., SÃ¡nchez, I.V., Zhu, M., Shah, S.D., Lee, W.W.	Y	Oral consent was gained before the interview and ppt received $20 voucher for their time. The project conforms with the Standards for Reporting Qualitative Research , and was approved as a quality improvement project by the University of Chicago. As such, it was deemed not human subjects research and was not reviewed by the Institutional Review Board.	Y	In-depth description of the analysis process, sufficient data are presented to support the findings, multiple people helped with the analysis reducing personal bias	Y	The researcher has discussed the credibility of their findings (triangulation, more than one analyst). The findings are discussed in relation to the original research question
Asgary, R. And Sckell, B. And Alcabes, A. And Naderi, R. And Adongo, P. And Ogedegbe, G.	Y	This study received IRB approval from the Lutheran Family Health Centres, New York City.	Y	There is an in-depth description of the analysis process, thematic analysis was used with clear info on how the categories/themes were derived from the data, 2 authors conducted the analysis reducing bias	Y	Adequate discussion of the evidence both for and against the researcher's arguments, the researcher has discussed the credibility of their findings (more than one analyst), the findings are discussed in relation to the original research question
Kim, H., Zhang, Y.	Y	Approved by the Human Subjects and Institutional Review Board at the University of Texas at Austin.	Y	There is an in-depth description of the analysis process, its clear how the categories/themes were derived from the data, the researcher explains how the data presented were selected from the original sample to demonstrate the analysis process, the researcher critically examined their own role, potential bias and influence during analysis and selection of data for presentation by testing inter-coder reliability.	Y	There is adequate discussion of the evidence both for and against the researcher's argument, the researcher has discussed the credibility of their findings (e.g. More than one analyst), the findings are discussed in relation to the original research question
Kramer, Justin and Yinusa-Nyahkoon, Leanne and Olafsson, Stefan and Penti, Brian and Woodhams, Elisabeth and Bickmore, Timothy and Jack, Brian W.	Y	All study protocols were approved by the BUSM Institutional Review Board and informed consent was obtained from all adult participants, with parental consent and participant assent having been obtained for those under the age of 18 years.	N	Lacks a lot of detail and doesn't say which method of analysis was used. But the researcher explains how the data presented were selected from the original sample to demonstrate the analysis process, contradictory data are taken into account, Whether the researcher reduced, potential bias and influence during analysis and selection of data for presentation by having 2 analysists.	Y	If there is adequate discussion of the evidence both for and against the researcher's arguments, the researcher has discussed the credibility of their findings (e.g. Triangulation more than one analyst), the findings are discussed in relation to the original research question
Maidment, D.W., Heyes, R., Gomez, R., Coulson, N.S., Wharrad, H., Ferguson, M.A.	Y	The study was approved by the NHS Health Research Authority, East of England Cambridgeshire and Hertfordshire Research Ethics Committee, and Nottingham University Hospitals NHS Trust Research and Innovation Department.	Y	There is an in-depth description of the analysis process, thematic analysis is used and is it clear how the categories/themes were derived from the data, the researcher explains how the data presented were selected from the original sample to demonstrate the analysis process, contradictory data are taken into account during teh analysis process, the researcher critically examined their own role, potential bias and influence during analysis and selection of data	Y	There is adequate discussion of the evidence both for and against the researcher's arguments, the researcher has discussed the credibility of their findings (e.g. More than one analyst), the findings are discussed in relation to the original research question

				for presentation by having a 2nd author work on the analysis as well		
Mizrachi, Y. And Shahrabani, S. And Nachmani, M. And Hornik, A.	Y	The ethics committee of the Max Stern Yezreel Valley College in Israel approved the current research (Emek no. 2012–17).	Y	Doesn't say what theory or process was used but there is an in-depth description of the analysis process, the researcher explains how the data presented were selected from the original sample to demonstrate the analysis process, sufficient data are presented to support the findings, the researcher critically examined their own role, potential bias and influence during analysis and selection of data for presentation	Y	There is adequate discussion of the evidence both for and against the researcher's arguments, the researcher has discussed the credibility of their findings (e.g. More than one analyst), the findings are discussed in relation to the original research question
Wikaire, E. And Harwood, M. And Wikaire-Mackey, K. And Crengle, S. And Brown, R. And Anderson, A. And Jansen, R. M. And Keenan, R.	Y	This study was categorised as low-risk (ie did not require full approval) by the New Zealand Health and Disability Ethics Committee review process. Additional ethical review was then completed and granted by the New Zealand Ethics Committee (NZEC)18 on 20 November 2020.	Y	Lacks a detail but does mention thematic analysis is used and is it clear how the categories/themes were derived from the data, the researcher explains how the data presented were selected from the original sample to demonstrate the analysis process, the researcher critically examined their own role, potential bias and influence during analysis and selection of data for presentation	Y	There is adequate discussion of the evidence both for and against the researcher's arguments, the findings are discussed in relation to the original research question
Yeong, J. L. And Thomas, P. And Buller, J. And Moosajee, M.	N	Only mentioned informed consent was provided by ppt, not mention of an ethics committee giving approval	N	Lacks detail and doesn't say which theory used to analyse. But the researcher explains how the data presented were selected from the original sample to demonstrate the analysis process	Y	There is adequate discussion of the evidence both for and against the researcher's arguments, the findings are discussed in relation to the original research question
Howells, K. And Amp, M. And Burrows, M. And Brown, J. And Brennan, R. And Dickinson, J. And Jackson, S. And Yeung, W. L. And Ashcroft, D. And Campbell, S. And Blakeman, T. And Sanders, C.	Y	Ethical approval was obtained from an NHS Research Ethics Committee (REC) committee.	Y	There is an in-depth description of the analysis process, thematic analysis was used and it is clear how the categories/themes were derived from the data, the researcher explains how the data presented were selected from the original sample to demonstrate the analysis process, sufficient data are presented to support the findings, the researcher critically examined their own role, potential bias and influence during analysis and selection of data for presentation	Y	There is adequate discussion of the evidence both for and against the researcher's arguments, the researcher has discussed the credibility of their findings (e.g. More than one analyst), the findings are discussed in relation to the original research question
Choix et al	Y	Mentions participants provided verbal informed consent. This study was IRB exempt.	N	Lacks details and uses minimal quotes	Y	Discussion of the evidence both for and against the researcher's arguments, the findings are discussed in relation to the original research question

Supplementary table 9: Participant demographics

Authors	CLEARS representation	Additional demographic details	Country
Alkureishi, et al. [39]	35 participants:<br>• **Culture (ethnicity):** Hispanic (n=2), Black/ African American (n=23), white (n= 8), other (n=2).<br>• **Educational attainment:** High school or less (n=14).<br>• **Socioeconomic status:** Medicare (n=25).	• 35 Adult primary care patients (27 female, 8 males) and 19 parents of paediatric patients who had a phone, video or both types of visits at a Chicago medical centre. Only the sample of 35 adult primary care patients were included in the analysis to avoid analysing paediatric perspectives.<br>• All reported English as their primary language.<br>• 10 in the analysed sample had higher economic status using medical plan as proxy (private medical insurance).<br>• 21 individuals has higher educational attainment (college/associate degree (n=7), Bachelor's degree (n=6), Graduate or professional degree (n=8)	Chicago (USA)
Asgary, et al. [27]	50 participants:<br>• **Residence (homeless):** Average years of homelessness: 2.03 yrs (max 14 yrs)	• 50 homeless individuals, of which 29 were female.<br>• 30 participants had a history of chronic diseases and 10 had an active mental illness.<br>• Sample had an average age: 51.66 and 33 were above the age of 50 but it is unclear how many were above 65.	New York City (USA)
Choxi et al. [40]	9 participants:<br>• **Culture (ethnicity):** Majority were Black, Indigenous, and People of Colour (BIPOC) (n=6)<br>• **Age:** Average age was 68, and a majority (n=5) were 70 or older.<br>• **Socioeconomic status (health insurance):** Majority had Medicaid or Medicare (n=7).	• 14 participants in total including older patients (n=9) and clinical support staff (n=4). Clinical support staff were excluded from analysis.	Portland, Oregon (USA)
Howells et al. [43]	21 participants:<br>• **Culture (ethnicity)**: mixed race (n=2), eastern European (n=1), white British (n=18)<br>• **Educational attainment:** no qualifications (n=11), GCSE (n=8), degree (n=1), missing (n=1)<br>• **Residence:** Homeless	• 43 participants of which 21 were homeless (including 3 females and 18 males) and 22 were healthcare professionals. Healthcare professionals were excluded from the analysis.<br>• All homeless individuals had at least one chronic condition and reported anxiety & depression and/ or alcohol & drug related issues.<br>• Homeless individuals age ranges included :25-34 (n=5), 35-44 (n=6), 45-54 (n=8), 55-64 (n=2)	Manchester (UK)
Kim, et al. [41]	20 articipants:<br>• **Culture (ethnicity and language):** All identified as Hispanic and 13 has spanish as their first language (n=13).<br>• **Educational attainment:** High school or less than high school education (n=15)<br>• **Socioeconomic status (income):** All low SES with 18 on an annual income of less than $25,000.	• A total of 20 participants of which 12 were female and 8 males.<br>• All reported having some kind of health-related concerns.<br>• Age of the sample ranged from 20 to 60.	Texas (USA)

Kramer et al. [42]	Boston (3 groups, 8 participants):<br>• **Culture (ethnicity):** black African American (n=5), black other (n=3)<br>• **Educational attainment**: high school (n=4), university (n=4)<br>• **Socioeconomic status (employment)**: unemployed (n=1), part time (n=4), full time (n=2), student (n=1)<br><br>Providence (3 groups and 19 participants):<br>• **Culture (ethnicity and language)**: black African American (n= 14), black other (n=5) and 3 prefer speaking Spanish at home.<br>• **Educational attainment**: no high school (n=4) less than high school (n=6), high school (n=4), university (n=5)<br>• **Socioeconomic status (employment):** unemployed (n=1), part time (n=3), full time (n=2), student (n=3)<br><br>New Haven (1 group and 12 participants):<br>• **Culture** (**ethnicity and language):** black African American (n=11), black other (n=1), and 3 prefer speaking Spanish at home.<br>• **Educational attainment:** no high school (n=1) less than high school (n=3), high school (n=4), university (n=4)<br>• **Socioeconomic status (employment):** Employment: part time (n=9), full time (n=3),<br><br>Philadelphia (2 groups, 9 participants):<br>• **Culture (ethnicity):** black African American (n=8), black other (n=1)<br>• **Educational attainment**: less than high school (n=4), high school (n=2) uni (n=3)<br>• **Socioeconomic status (employment):** unemployed (n=3), part time (n=1), full time (n=0), student (n=5)	• 67 in focus group and 5 in interviews (12 key informant interviews excluded and 19 minors (under 18) excluded from analysis).<br>• Interviewees were recruited from the sample of 30 men who used Embodied conversational agent for 2 weeks.<br>• Mean ages of recruitment sites:<br>○ Boston: 23.4yrs<br>○ Providence: 21.6yrs<br>○ New Haven: 24yrs<br>○ Philadelphia: 19.8yrs	Boston, New Haven, Providence, & Philadelphia (USA)
Maidment et al. [44]	16 participants:<br>• **Limiting conditions**: All had a hearing impairment.<br>**Age range**: 39-85 (mean: 68.81)	• 16 first time hearing aid users recruited through a medical centre in Nottingham.<br>• 6 were female and 10 were males.<br>• 2 self-reported as a beginner in digital tech competency and 14 as fully competent.<br>• All participants had a good understanding of the English language.	Nottingham (UK)
Mizrachi, et al. [46]	31 participants:<br>• **Culture (religion):** Jewish (n= 25), other religion (n=6)<br>• **Age:** 61 and 69 (n=7), 70 and above (n=6).	• 31 people picked from the sample the authors used in a quantitative survey in a different study.<br>• 21 users of online health services (9 men and 12 women) and 10 non-users (4 men and 6 women).<br>• Other ages included between 50 and 54 (n=12), 55 and 60 (n=6),	Israel
Wikaire, et al. [47]	12 participants:<br>• **Culture (ethnicity):** native New Zealand individuals	• 6 Māori telehealth consultation patients and 6 Māori in-clinic consultation patients. (5 Māori	New Zealand

		health professionals were excluded from analysis).	
Yeong, et al. [45]	9 participants:<br>• **Limiting conditions**: Visually impaired (n=6),<br>• **Age:** above 65 (n=1)<br>• **Socioeconomic status:** Unemployed (n=1)	• 4 females and 5 males who all used the internet and all reported to be at least somewhat confident in using tech. Some healthcare professionals were interviewed but are excluded from analysis.	London (UK)

Supplementary table 10: Included articles details

**Author**	**Aim**	**Method**	**Inclusive digital health strategy**	**Factor(s) causing inequities**	**Overarching theme covered**
Alkureishi, et al. [39]	To understand patients' perspectives on (1) the definition, causes, and impact of the digital divide; (2) whose responsibility it is to address the digital divide; and (3) potential solutions to mitigate the digital divide.	Interviews	Co-designing general digital health services	Older, low-income, minority, and non-English speaking Medicaid patients are at highest risk of experiencing technology access and digital literacy barriers.	• Infrastructure<br>• Educational support
Asgary, et al. [27]	To evaluate homeless individuals' perceptions, attitudes and experiences regarding mobile health.	Interviews	Co-designing mHealth	Little information exists regarding strategies that use mHealth to mitigate barriers to healthcare access among the homeless.	• Infrastructure<br>• User-friendly design
Choxi et al. [40]	Identify the barriers of video visits for patients who rely on telephone visits for ambulatory care.	Interviews	Co-designing video consultation health services	Due to covid, video visits increased to allow physical examination and aid diagnostic decision making but require video capable technology, reliable internet access and greater digital literacy than phone visits contributing towards inequities.	• Educational support<br>• User-friendly design
Howells et al. [43]	Explore the experience and impact of organisational and technology changes in response to COVID-19 on access to health care for people experiencing homelessness.	Interviews	Outreach	Little is known about whether the recent changes required during COVID-19 to deliver services to people during experiencing homelessness will help to address or compound inequality in accessing care.	• Infrastructure<br>• Educational support
Kim, et al. [41]	Exploring the context in which	Interviews	Co-designing mHealth	Little is known about the users, particularly those of	• Infrastructure

	smartphones were used for health information.			low-socioeconomic status, health information-seeking behaviour using smartphones.	• User-friendly design
Kramer et al. [42]	Black men's experiences with, and suggestions for, health care systems, while also exploring the acceptability of ECA technology (embodied conversational agent (ECA) that simulates face-to-face conversation with a health counsellor).	Interviews & focus groups	Co-designing Embodied conversational agent (ECA)	Black individuals are more likely to live in medically underserved areas, technology may offer an opportunity to address health disparities by improving trust & cultural sensitivity.	• User-friendly design
Maidment et al. [44]	Compare barriers and facilitators between m2Hear (app which provides information on how to use a hearing aid) and C2Hear (YouTube videos delivering similar information as m2Hear).	Interviews	Co-designing mHealth	First-time users experience difficulties using their hearing aids because they struggle to remember all of the information given to them by their audiologist at the time of fitting. An app could provide easily accessible information to promote proper use.	• Educational support<br>• User-friendly design
Mizrachi, et al. [46]	Explore the role of family members in supporting online health services usage amongst older groups.	Interviews	Outreach	Low adoption of online health services amongst over 65s.	• Educational support<br>• User-friendly design
Wikaire, et al. [47]	Investigate Māori (New Zealand natives) experiences of Telehealth consultations during the March 2020 COVID-19 lockdown.	Interviews	Co-designing General digital health services	Telehealth may present additional barriers to) that contribute to inequities.	• Infrastructure
Yeong, et al. [45]	Identify key web-based accessibility features for internet users with vision impairment and to explore whether the contents provided in website were relevant and comprehensible.	Interviews & focus groups	Co-designing Health websites	Health websites for genetic eye disorders are typically poorly accessible to users with visual impairment.	• User-friendly design
